# Fabrication of three-dimensionally interconnected nanoparticle superlattices and their lithium-ion storage properties

**DOI:** 10.1038/ncomms7420

**Published:** 2015-03-03

**Authors:** Yucong Jiao, Dandan Han, Yi Ding, Xianfeng Zhang, Guannan Guo, Jianhua Hu, Dong Yang, Angang Dong

**Affiliations:** 1 State Key Laboratory of Molecular Engineering of Polymers and Department of Macromolecular Science, Fudan University, Shanghai 200433, China; 2 Department of Chemistry and Collaborative Innovation Center of Chemistry for Energy Materials, Fudan University, Shanghai 200433, China

## Abstract

Three-dimensional superlattices consisting of nanoparticles represent a new class of condensed materials with collective properties arising from coupling interactions between close-packed nanoparticles. Despite recent advances in self-assembly of nanoparticle superlattices, the constituent materials have been limited to those that are attainable as monodisperse nanoparticles. In addition, self-assembled nanoparticle superlattices are generally weakly coupled due to the surface-coating ligands. Here we report the fabrication of three-dimensionally interconnected nanoparticle superlattices with face-centered cubic symmetry without the presynthesis of the constituent nanoparticles. We show that mesoporous carbon frameworks derived from self-assembled supercrystals can be used as a robust matrix for the growth of nanoparticle superlattices with diverse compositions. The resulting interconnected nanoparticle superlattices embedded in a carbon matrix are particularly suitable for energy storage applications. We demonstrate this by incorporating tin oxide nanoparticle superlattices as anode materials for lithium-ion batteries, and the resulting electrochemical performance is attributable to their unique architectures.

## Abstract (web summary)

Three-dimensional nanoparticle superlattices are interesting, but their fabrication is generally limited to materials attainable as monodisperse colloidal nanoparticles. Here, the authors fabricate interconnected superlattices via self-assembly without the presynthesis of the constituent nanoparticles.

## Graphical abstract

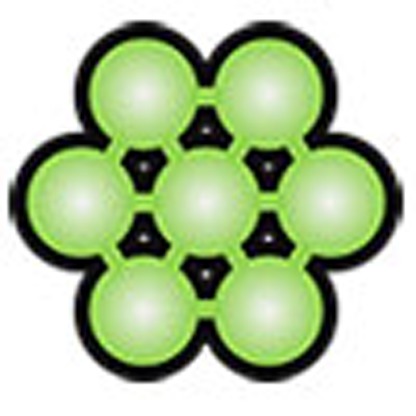

Three-dimensional (3D) superlattices consisting of nanoparticles (NPs) are emerging as a new and important class of nanostructured materials^1^, the properties of which can be rationally tuned by manipulating the size, shape and composition of the constituent NPs. In particular, interparticle interactions in NP superlattices can lead to collective properties, which are significantly different from the isolated NPs^1^,^2^,^3^. Existing methods of growing NP superlattices rely on the self-assembly of monodisperse colloidal NPs induced by solvent evaporation or antisolvent destabilization^1^. Recent progress on the self-assembly of NPs has been witnessed by the growth of a rich array of single- and multicomponent NP superlattices^4^,^5^,^6^,^7^,^8^,^9^,^10^, which have found wide applications in electronic and optoelectronic devices, catalysis and energy storage.

The self-assembly of colloidal NPs is a complicated process, involving various driving forces and interactions (for example, van der Waals, Coulombic, dipolar)^1^. As a consequence, successful assembly of long-range ordered NP superlattices requires exquisite control over many parameters, such as NP size distribution, surface-coating ligands and solvent evaporation kineticsand so on. In addition, self-assembled NP superlattices typically suffer from poor electrical conductivity due to the large interparticle spacing maintained by the capping ligands^1^,^11^. Therefore, post-surface treatment such as ligand exchange is required to enhance electronic coupling, which unfortunately can lead to severe structural defects such as cracks^12^,^13^. Another major bottleneck hampering prospects of NP superlattices is the limited choice of monodisperse NP building blocks, despite recent progress in colloidal synthesis^1^,^14^.

In this work we report an approach that can overcome the aforementioned limitations associated with self-assembly methods, enabling the growth of 3D interconnected, strongly coupled NP superlattices without the presynthesis of the constituent NPs. The resulting connected NP superlattices embedded in 3D continuous carbon frameworks represent a new class of superlattice materials, which are demonstrated to be remarkably promising for energy storage applications. SnO_2_ NP superlattices are chosen as a model system to study Li-ion storage properties, which exhibit exceptional cycling stability and rate capability when used as Li-ion battery (LIB) anodes.

## Results

### Fabrication procedure

**Figure 1:**
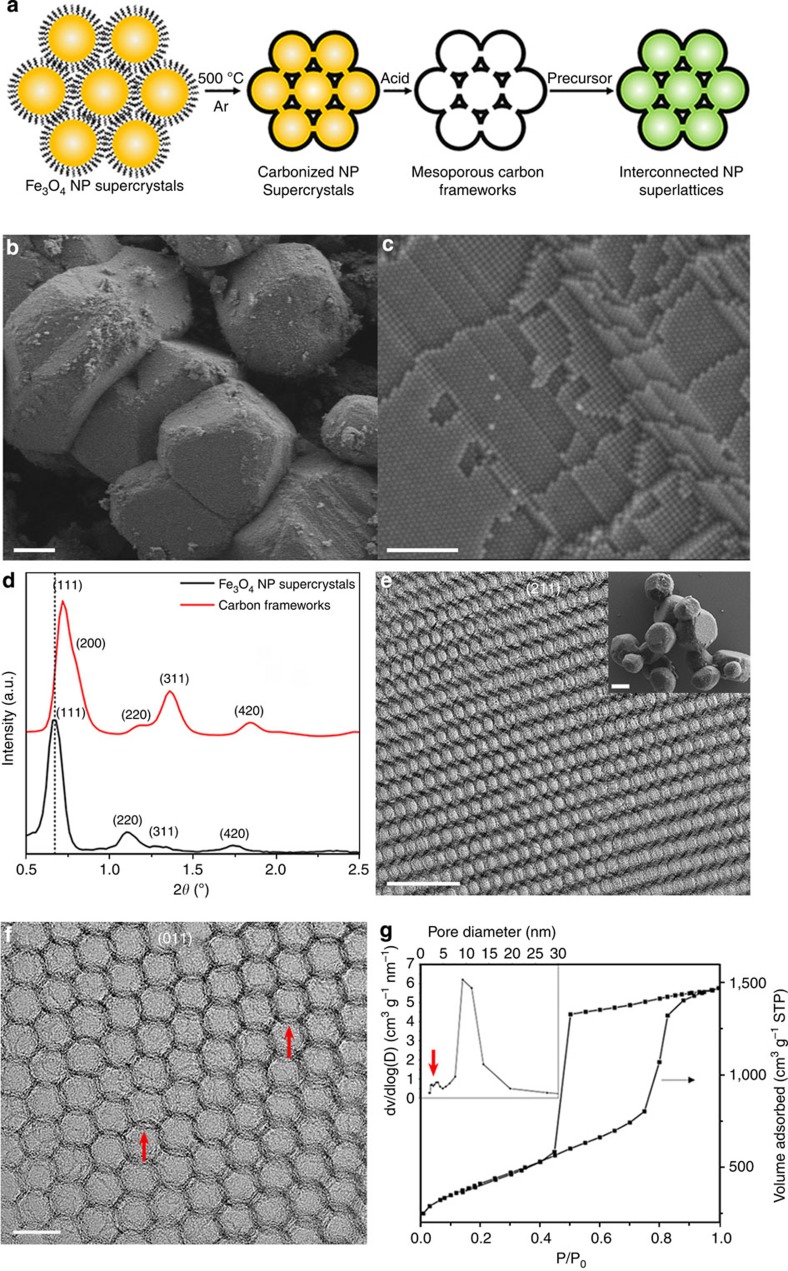
Fabrication of three-dimensionally interconnected NP superlattices from mesoporous carbon frameworks. (**a**) Schematic illustration of the fabrication procedure (cross-sectional view). (**b**,**c**) SEM and HRSEM images of carbonized Fe_3_O_4_ NP supercrystals, respectively. Scale bars, 1 μm and 200 nm, respectively. (**d**) SAXS patterns of carbonized Fe_3_O_4_ NP supercrystals and mesoporous carbon frameworks, respectively. (**e**,**f**) TEM images of mesoporous carbon frameworks with different lattice projections. Scale bars, 50 and 20 nm, respectively. The inset in (**e**) is a low-magnification SEM image of mesoporous carbon frameworks. Scale bar, 1 μm. The red arrows in (**f**) indicate the interconnected windows. (**g**) N_2_ adsorption–desorption isotherms and the corresponding pore size distribution (inset) of mesoporous carbon frameworks. The red arrow indicates the small pores corresponding to the interconnected windows observed in (**f**).

Figure 1a schematically illustrates the overall fabrication procedure. Briefly, starting with self-assembled Fe_3_O_4_ NP supercrystals, we obtain 3D continuous, highly ordered mesoporous carbon frameworks on the carbonization of the surface-coating oleic acid (OA) ligands followed by the removal of Fe_3_O_4_ NPs. The subsequent impregnation of carbon frameworks with desired precursors leads to 3D NP superlattices on hydrolysis and/or thermal annealing. The constituent NP composition can be modulated by selecting appropriate precursors, while the NP size can be tailored by controlling that of the original Fe_3_O_4_ NPs. The reason to select Fe_3_O_4_ NPs as the starting material is that the scalable production of Fe_3_O_4_ NP superlattices can be routinely achieved by self-assembly^14^,^15^. In addition, Fe_3_O_4_ is an inexpensive and thermally stable material, which can be readily removed by acid etching.

It is worth to mention that although the structure of our mesoporous carbon frameworks appears to be similar to that of previous mesoporous carbons prepared from hard template (that is, silica opals or mesoporous silica)^16^,^17^,^18^,^19^,^20^,^21^,^22^, we should emphasize that this is the first preparation of ordered mesoporous carbons from inorganic nanocrystals. There are several unique features associated with this preparation strategy, leading to mesoporous carbons with distinctive structural and textural properties. First, the surface-coating OA ligands, which are a necessary component for the growth and self-assembly of Fe_3_O_4_ NPs, concurrently serve as the carbon source, yielding mesoporous carbons having ultrathin pore walls (~2 nm) and the same topological structure as the Fe_3_O_4_ NPs supercrystal template. This is in sharp contrast with previous hard-templating approaches where additional carbon precursors (that is, polymers or prepolymers) have to be applied to fill the voids of the template and the resulting mesoporous carbons usually possess an inverse structure of the template^16^,^17^,^18^,^19^,^20^,^21^,^22^. Furthermore, unlike previous approaches where silica opals or mesoporous silica only act as template^16^,^17^,^18^,^19^,^20^,^21^,^22^, the Fe_3_O_4_ NPs used here also serve as a graphitization catalyst (to be discussed below), allowing for partially graphitic frameworks at low temperatures (500 °C). More importantly, the unique structure of our carbon frameworks enables interconnected, strongly coupled NP superlattices, which could not be readily accessible by conventional self-assembly methods.

### Mesoporous carbon frameworks

Monodisperse Fe_3_O_4_ NPs (~11 nm) stabilized by OA are synthesized by a literature method^14^ (Supplementary Fig. 1a), and Fe_3_O_4_ NP superlattices are grown by a conventional drying-mediated self-assembly process (Supplementary Fig. 1b)^4^. The content of the surface-coating OA ligands determined from thermogravimetric analysis (TGA) is~15.9 wt% (Supplementary Fig. 1c), corresponding to the area ligand coverage of~3.83 nm^−2^ (that is,~1455 OA molecules per NP, Supplementary Note 1). The prior addition of squalane (~1 wt%) into the NP solution is found to favour the gram-scale growth of micrometer-sized NP supercrystals with well-developed facets^15^, although squalane is not critical for the subsequent formation of mesoporous carbon frameworks (Supplementary Fig. 1d). The as-assembled NP supercrystals are then heated at 500 °C in an argon atmosphere, producing grey powders arising from the carbonization of OA (Supplementary Fig. 2a). The carbon species constitute~11 wt% of the carbonized NP supercrystals, determined from TGA (Supplementary Fig. 2b). Scanning electron microscopy (SEM) and high-resolution SEM (HRSEM) establish that both the faceted morphology and the long-range NP ordering are well retained in carbonized Fe_3_O_4_ NP supercrystals (Fig. 1b,c and Supplementary Fig. 2c–f). These results strongly suggest that cracks and other structural defects, which have been observed in many thermally treated NP superlattices reported previously^23^,^24^, are largely prevented in our experiments, probably due to the 3D crystal-like morphology as well as the long-range structural ordering of our Fe_3_O_4_ NP supercrystals. The small-angle X-ray scattering (SAXS) pattern of carbonized Fe_3_O_4_ NP supercrystals exhibits at least four scattering peaks, which can be assigned to the 111, 220, 311, 420 reflections of a well-crystallized face-centered cubic (fcc) structure (Fig. 1d, black curve), consistent with the highly ordered superlattice structure observed in HRSEM (Fig. 1c and Supplementary Fig. 2f). The unit cell parameter calculated from the SAXS data is 22.8 nm.

HNO_3_ or HCl etching is employed to treat carbonized NP supercrystals. The complete removal of Fe_3_O_4_ NPs yields black carbon powders after washing and drying (Supplementary Fig. 3a), which display a highly ordered porous structure as revealed by SEM (Supplementary Fig. 3b) and transmission electron microscopy (TEM, Fig. 1e,f and Supplementary Fig. 3c,d). SAXS (Fig. 1d, red curve) and SEM (Fig. 1e, inset) indicate that the porous carbon frameworks possess the same fcc structure and faceted morphology inherited from Fe_3_O_4_ NP supercrystals, while the pore size (~10 nm) is slightly smaller than the diameter of Fe_3_O_4_ NPs, probably caused by the framework shrinkage during etching and/or post drying processes. Interestingly, high-resolution TEM (HRTEM) and Raman spectroscopy suggest that the pore walls of carbon frameworks are partially graphitic (Supplementary Fig. 4), which is remarkable considering the low carbonization temperature (500 °C). We attribute the formation of partially graphitic frameworks at such a low temperature to the use of Fe_3_O_4_ NPs, as transition metals such as Fe and Co and the corresponding metal oxides have been widely used as graphitization catalysts^25^,^26^,^27^. We surmise that the carbonaceous species arising from the thermal decomposition of OA molecules leads to the partial reduction of Fe_3_O_4_ NPs (most likely surface atoms) into metallic iron, which concurrently promotes the graphitization of the surrounding carbonaceous layers into partially graphitic pore walls. Despite the thin wall thickness (~2 nm, Supplementary Fig. 4b), the graphitization degree of carbon frameworks can be further increased by heat treatment at 1,200 °C in argon, converting the pore walls into few-layer graphene while retaining structural ordering (Supplementary Fig. 4c). More interestingly, tilted experiments along the [011] zone axis in TEM clearly shows that the adjacent pores are connected through small windows with dimensions of~3 nm (Fig. 1f, indicated by red arrows), leading to a 3D continuous porous structure. Presumably, the formation of the interconnected windows is attributed to the slight sintering of neighbouring Fe_3_O_4_ NPs occurring during the carbonization process.

The porous structure of carbon frameworks is further characterized by N_2_ adsorption–desorption isotherms, which show a type-IV curve with a sharp capillary condensation step occurring in the relative pressure (P/P_0_) range of 0.8–0.85 (Fig. 1g), a typical feature of mesoporous materials^28^. The Brunauer–Emmett–Teller surface area and the pore volume are determined to be ~1,500 m^2^ g^−1^ and ~2.5 cm^3^ g^−1^, respectively. The pore size distribution curve determined by using the Barrett–Joyner–Halenda model suggests a bimodal porous structure (Fig. 1g, inset). The large pores at ~10 nm correspond to the removed Fe_3_O_4_ NPs, while the small pores in the range of 2–4 nm are ascribed to the interconnected windows observed in TEM.

### Interconnected NP superlattices

**Figure 2:**
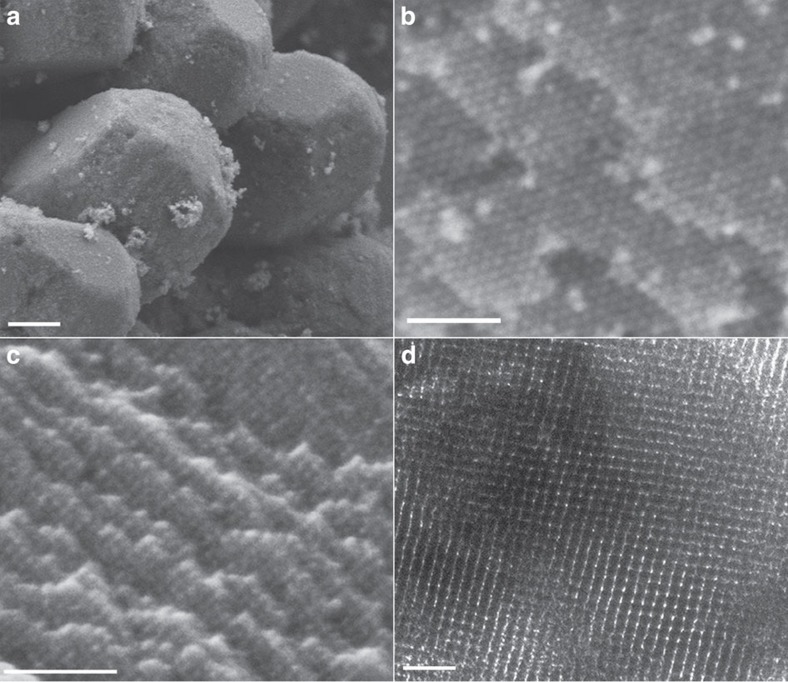
Representative electron microscopy images of various NP superlattices. (**a**) SEM image of SiO_2_ NP superlattices, showing the faceted morphology. Scale bar, 500 nm. (**b**) HRSEM image of SiO_2_ NP superlattices, showing the surface terraces and long-range NP ordering. Scale bar, 100 nm. (**c**,**d**) SEM and TEM images of 3D carbon NP superlattices, respectively. Scale bars, 100 and 50 nm, respectively.

The 3D continuous porosity and high surface area of carbon frameworks are expected to facilitate the diffusion of precursors, which is crucial for the subsequent growth of NP superlattices. As a proof-of-concept demonstration, tetraethoxysilane is chosen as the precursor for SiO_2_ NP superlattices, which are obtained by repeated precursor infiltration followed by hydrolysis. Remarkably, detailed structural features such as facets and surface terraces originated from Fe_3_O_4_ NP supercrystals are also observed in the product (Fig. 2a,b and Supplementary Fig. 5), indicating a high degree of 3D NP ordering. The flexibility offered by the approach and the robustness of carbon frameworks allow the ready tuning of NP compositions by simply selecting appropriate precursors. For example, pure carbon NP superlattices can be realized by impregnating carbon frameworks with an aqueous solution of sucrose followed by drying and calcination (Fig. 2c,d). Energy-dispersive X-ray spectroscopy (EDS) and elemental mapping confirm that the resulting superlattices are composed of carbon NPs (Supplementary Fig. 6). To our knowledge, this is the first preparation of 3D carbon NP superlattices, as monodisperse, ligand-capped carbon NPs are not yet accessible for self-assembly^29^.

**Figure 3:**
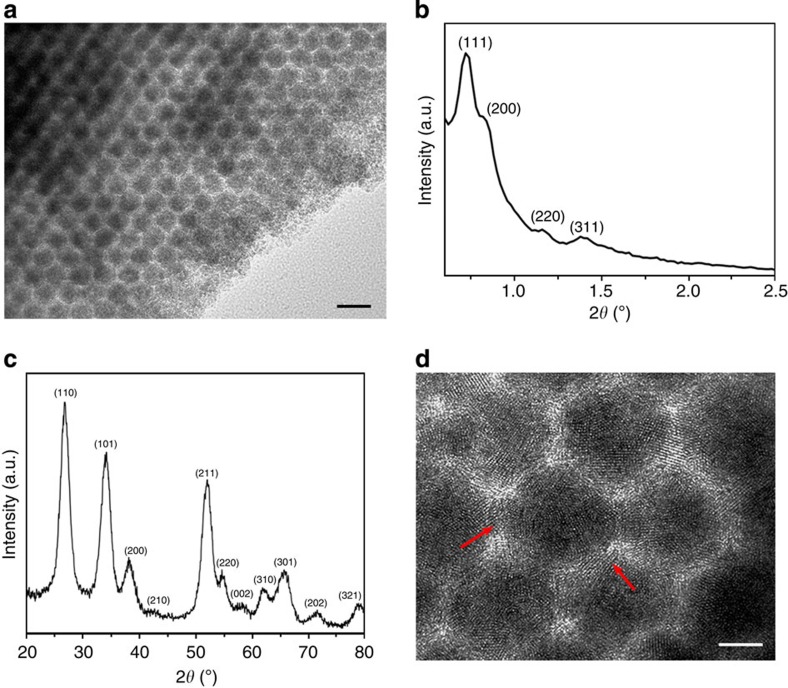
Structural characterization of SnO_2_ NP superlattices. (**a**) TEM image of SnO_2_ NP superlattices. Scale bar, 20 nm. (**b**,**c**) SAXS pattern and XRD pattern of SnO_2_ NP superlattices, respectively. (**d**) HRTEM image of SnO_2_ NP superlattices, showing the high crystallinity of the embedded SnO_2_ NPs. Scale bar, 5 nm. The red arrows indicate NP interconnections.

To further illustrate the generality of the approach, we fabricate superlattices of crystalline metal oxide NPs such as SnO_2_ by using SnCl_2_ as the precursor. Thermal annealing at 350 °C in argon is carried out to crystallize the embedded NPs. TEM suggests that the crystallization process does not disrupt the ordered structure of SnO_2_ NP superlattices (Fig. 3a and Supplementary Fig. 7), while SAXS confirms the fcc geometry with a lattice constant of 21.2 nm (Fig. 3b). Powder X-ray diffraction (XRD, Fig. 3c) and HRTEM (Fig. 3d) establish that the constituent SnO_2_ NPs possess a high crystallinity with a tetragonal crystal structure. In addition to SnO_2_, other types of metal oxide nanocrystal superlattices can be prepared in a similar way using metal alkoxides or anhydrous inorganic salts as precursors. For instance, 3D superlattices consisting of anatase TiO_2_ nanocrystals are formed by impregnating carbon frameworks with titanium isopropoxide (TIP) followed by hydrolysis and thermal annealing (Supplementary Fig. 8). Moreover, NP superlattices of multicomponent phases, such as mixed oxides and metal phosphates, are also accessible by using a pre-mixed precursor (Supplementary Fig. 9). Apparently, the NP composition can be tailored by changing the ratio of the two precursors. For example, Ti_0.3_Sn_0.7_O_2_ NP superlattices are obtained when a mixture of SnCl_2_ and TIP is employed as the precursor, and the uniform distribution of Ti and Sn as revealed by elemental mapping indicates that the homogenous filling of Ti_0.3_Sn_0.7_O_2_ NPs within the carbon framework (Supplementary Fig. 9c–e). The ability to fabricate such multiphase NP superlattices is particularly important, as they are generally hard to be prepared by self-assembly due to the challenge to obtain the corresponding monodisperse NPs.

### Li-ion storage properties

One unique structural feature of NP superlattices produced by our approach is that the neighbouring NPs are connected with each other through interconnected windows within carbon frameworks, as shown in Fig. 3d where SnO_2_ NP connections can be clearly observed (indicated by red arrows). Such connection configuration could substantially enhance electronic coupling between close-packed NPs^30^,^31^, which is highly desirable for applications requiring high electrical conductivity. In comparison to self-assembled NP superlattices, another important feature of our NP superlattices is that the constituent NPs are embedded in a continuous and partially graphitic carbon matrix. Such carbon coating could further facilitate electron transport and accommodate volume variations of NPs, which is particularly beneficial for energy storage devices^32^,^33^,^34^. To demonstrate this, SnO_2_ NP superlattices are chosen as anode materials for LIBs, which exhibit excellent electrochemical performance in terms of cycling stability and rate capability. The reason to select SnO_2_ NP superlattices is that SnO_2_ has attracted increasingly greater attention for Li-ion storage due to its high theoretical capacity (780 mAh g^−1^) and environmental benignity^35^,^36^,^37^,^38^,^39^,^40^,^41^,^42^,^43^,^44^.

**Figure 4:**
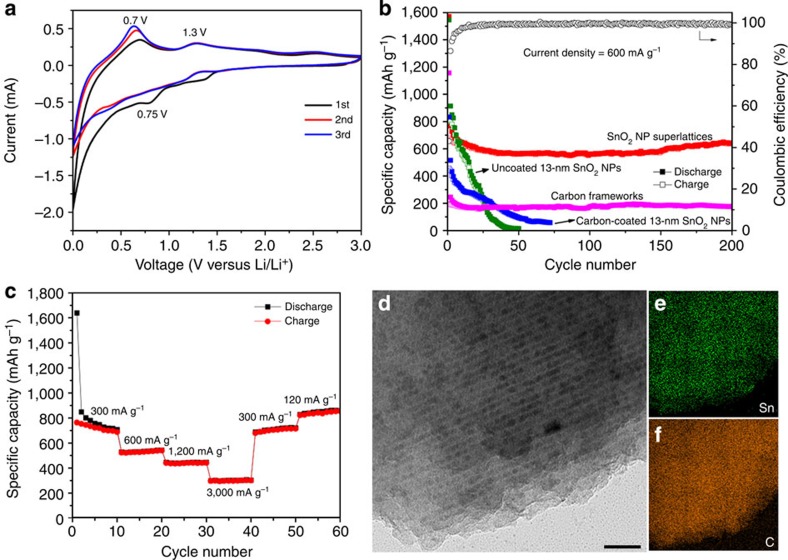
Electrochemical characterization of SnO_2_ NP superlattices. (**a**) Representative cyclic voltammograms at a scan rate of 0.5 mV s^−1^. (**b**) Cycling performance at a current density of 600 mA g^−1^ and the corresponding Coulombic efficiency. The cycling performance of 13-nm SnO_2_ NPs with and without carbon coating as well as carbon frameworks tested under the same conditions are also included for comparison. (**c**) Rate-capability test at current densities ranging from 120 to 3,000 mA g^−1^. (**d**) TEM image and (**e**,**f**) the corresponding EDS elemental mapping of SnO_2_ NP superlattices after 200 cycles, showing the preservation of ordered structure without NP aggregation. Scale bar, 50 nm.

To investigate their electrochemical performance, LIB anodes based on SnO_2_ NP superlattices are cycled on the basis of the half-cell configuration. The carbon content of SnO_2_ NP superlattices is ~28 wt%, determined from TGA (Supplementary Fig. 10a). Electron microscopies indicate that SnO_2_ NP superlattices are uniformly distributed in the electrode with well-retained structural ordering before cycling (Supplementary Fig. 10b–d). Fig. 4a shows the cyclic voltammograms of SnO_2_ NP superlattices in the potential range from 3.0 to 0.005 V (versus Li/Li^+^) at a scan rate of 0.5 mV s^−1^. The irreversible peak at 0.75 V in the first lithiation process is attributed to the reduction of SnO_2_ into Sn as well as the formation of a solid electrolyte interphase (SEI)^39^. The two peaks at 0.7 and 1.3 V in the first delithiation process correspond to the de-alloying of Li_*x*_Sn and the partially reversible reaction of SnO_2_ with Li^+^, respectively, consistent with previous results^37^,^38^. The cycling stability of SnO_2_ NP superlattices is studied by galvanostatic charge/discharge. The first discharge process leads to a capacity of 1,570 mAh g^−1^ at a relatively high current density of 600 mA g^−1^, whereas the subsequent charge process delivers a capacity of 676 mAh g^−1^ (Supplementary Fig. 11). This irreversibility is probably caused by the SEI formation as well as the lithium insertion into carbon frameworks^36^. Despite the first-cycle capacity loss commonly observed for SnO_2_ anodes^36^,^37^, our SnO_2_ NP superlattices exhibit excellent cycling stability, as manifested by the retention of a specific capacity of 640 mAh g^−1^ after 200 cycles and a stabilized Coulombic efficiency over 98% from the 10th cycle (Fig. 4b). LIB anodes based on mesoporous carbon frameworks only are also tested under the same conditions, which exhibit a stable capacity at 185 mAh g^−1^ after 200 cycles (Fig. 4b, pink curve), indicating that the high capacity of our SnO_2_ NP superlattices is primarily attributed to the embedded SnO_2_ NPs. To better evaluate the charge/discharge performance of our SnO_2_ NP superlattices, we synthesize colloidal SnO_2_ NPs with a similar diameter (~ 13 nm, Supplementary Fig. 12)^45^, which are cycled under the same conditions in control experiments. As expected, the uncoated SnO_2_ NPs exhibit fast capacity decay within 50 cycles (Fig. 4b, olive curve), presumably caused by the pulverization and/or aggregation of SnO_2_ NPs in the absence of carbon protection. In comparison, the carbon-coated SnO_2_ NPs (carbon content:~26 wt%) exhibit better cycling stability, but their capacity rapidly fades below 100 mAh g^−1^ after 50 cycles (Fig. 4b, blue curve). The cycling performance of our SnO_2_ NP superlattices is further evaluated at different current densities. As shown in Fig. 4c, the electrode is able to deliver a capacity of 300 mAh g^−1^ even at a current density of 3,000 mA g^−1^, and a capacity above 850 mAh g^−1^ is recovered when the current density is switched back to 120 mA g^−1^. Such cycling stability and rate capability outperform those of most SnO_2_ NP anodes reported previously^38^,^39^,^40^,^41^,^42^,^43^,^44^.

## Discussion

The superior battery performance of SnO_2_ NP superlattices is attributed to their unique structural characteristics. First, our NP superlattices exist as micrometer-sized secondary particles inherited from the original Fe_3_O_4_ NP supercrystals, which are believed to be the ideal architectures for LIBs due to the reduced interfacial areas between active materials and electrolyte^46^,^47^. Second, and perhaps most importantly, the 3D graphitic carbon frameworks combined with interconnected NPs provide a continuous electron pathway, facilitating electronic connectivity within the components of the electrode. Moreover, the inherent flexibility of carbon frameworks can buffer large volume expansions of the embedded SnO_2_ NPs during cycling^35^,^36^, reducing the strain of the entire electrode.

To investigate the structural evolution of our SnO_2_ NP superlattices during cycling, ex situ XRD, SEM, TEM, and EDS elemental mapping studies are performed after 200 cycles. In accordance with previous results for SnO_2_-based anodes^35^,^43^, XRD confirms the conversion of SnO_2_ NPs into Sn NPs after cycling (Supplementary Fig. 13a), while the broad diffraction peaks imply that the resulting Sn NPs are small in size without agglomeration. SEM reveals that the secondary particle morphology is largely preserved after cycling (Supplementary Fig. 13b,c), while TEM confirms that the Sn-based NPs are homogeneously confined within carbon frameworks with good structural ordering without aggregation (Fig. 4d–f and Supplementary Fig. 13d). These results strongly suggest that our carbon frameworks effectively suppress the agglomeration and pulverization of Sn-based NPs, which could explain the superior cycling stability of our SnO_2_ NP superlattices. It should also be noted that the LIB performance could be further improved by enhancing the electrical conductivity of carbon frameworks before precursor infiltration, which can be realized by increasing the graphitization degree via heat treatment as mentioned above.

In summary, we have developed a new approach to fabricate three-dimensionally interconnected, strongly coupled NP superlattices with fcc packing symmetry. The generality of the approach is illustrated by the growth of NP superlattices with various compositions including oxides, carbon, mixed oxides, and phosphates. We also demonstrate the successful use of SnO_2_ NP superlattices as anode materials for LIBs, and the excellent cycling stability and rate capability are attributed to their unique architectures (that is, 3D continuous and graphitic carbon coating, NP interconnections, and supercrystal morphology). Given the fact that the fabrication procedure does not require the presynthesis of monodisperse NP building blocks, we anticipate that in the future a wider range of materials can be prepared as interconnected NP superlattices, which can find various applications in electronics, catalysis, and energy storage.

## Methods

### Materials

Oleic acid (OA, 90%), squalane, and 1-octadecene (ODE, 90%) were purchased from Aldrich. Sodium oleate was obtained from TCI. Iron chloride hexahydrate (FeCl_3_.6H_2_O), titanium isopropoxide (TIP), anhydrous tin chloride (SnCl_2_), anhydrous tin tetrachloride (SnCl_4_) tetraethoxysilane (TEOS), anhydrous zirconium tetrachloride (ZrCl_4_), Cetyl trimethyl ammonium bromide (CTAB), and triethyl phosphate were purchased from Aladdin. All chemicals were used as received without further purification.

### Synthesis and self-assembly of Fe_3_O_4_ NPs

Monodisperse, OA-stabilized Fe_3_O_4_ NPs with a diameter of~11 nm were synthesized according to the literature method^14^. In a typical synthesis, 72 g of iron oleate and 11.4 g of OA were dissolved in 400 g of ODE in a three-neck flask, and the resulting solution was heated at 320 °C under a N_2_ atmosphere for 1 h. After cooling down to room temperature, ethanol and isopropanol were added to precipitate Fe_3_O_4_ NPs, and the precipitates were dispersed in hexane to form a stable colloidal solution with a concentration of~10 mg ml^−1^. To self-assemble Fe_3_O_4_ NP supercrystals, squalane (~ 1 wt%) was added to the NP solution and the solvent was then allowed to evaporate under ambient conditions. The complete solvent evaporation yielded faceted Fe_3_O_4_ NP supercrystals with dimensions on the micrometer scale.

### Fabrication of three-dimensionally interconnected NP superlattices

The as-assembled Fe_3_O_4_ NP supercrystals were heated in a quartz tube furnace at 500 °C for 2 h in argon, converting the surface-coating ligands into carbon. The carbonized NP supercrystals were then refluxed in a HNO_3_ or HCl solution to remove the embedded Fe_3_O_4_ NPs. The resulting mesoporous carbon frameworks retrieved by centrifugation were washed with deionized water, yielding black powders after drying. To grow interconnected NP superlattices, the dried porous carbon frameworks were infiltrated with desired precursors via wet impregnation followed by hydrolysis and/or thermal annealing.

### Preparation of SiO_2_ NP superlattices

5 mg of dried mesoporous carbon powder was dissolved in 1 ml of TEOS. After stirring for 6 h, the precipitated powder collected by centrifugation was washed with ethanol to remove the excess TEOS. The hydrolysis of TEOS was induced by the addition of ammonia hydroxide, leading to the conversion of TEOS into SiO_2_. This impregnation/washing cycle was repeated twice in order to completely fill carbon frameworks with SiO_2_ NPs.

### Preparation of carbon NP superlattices

3D carbon NP superlattices were synthesized using sucrose as the precursor. Briefly, 0.5 g of sucrose and 0.1 g of concentrated H_2_SO_4_ were first dissolved in 1 ml of H_2_O to form a mixture, into which 5 mg of dried mesoporous carbon powder was added with constant stirring. After 2 h, the precipitated powder collected by centrifugation was washed with H_2_O to remove the excess sucrose and H_2_SO_4_. After that, the product was placed in an oven at 100 °C for 2 h and then 160 °C for another 2 h. The black powder was then impregnated in a diluted sucrose solution (0.05 g of sucrose, 0.02 g of H_2_SO_4_, and 1 ml of H_2_O) for 2 h. After washing and drying, the product was heated at 600 °C in argon for 2 h to carbonize sucrose, yielding 3D carbon NP superlattices.

### Preparation of SnO_2_ NP superlattices

50 mg of mesoporous carbon powder was mixed with a SnCl_2_ solution dissolved in ethanol (0.1 g ml^−1^) and the resulting suspension was stirred for 2 h. After that, the black powder was collected by centrifugation and the precipitated powder was washed with ethanol to remove the excess SnCl_2_. The precursor hydrolysis was initiated by the addition of ammonium hydroxide. This impregnation/washing cycle was repeated twice in order to completely load carbon frameworks with SnO_2_. Thermal annealing at 350 °C in argon for 1 h was carried out to crystallize the embedded SnO_2_ NPs.

### Preparation of TiO_2_ NP superlattices

TIP was used as the precursor for the fabrication of TiO_2_ NP superlattices. Briefly, 50 mg of dried mesoporous carbon powder was mixed with a TIP solution dissolved in isopropanol (0.5 g ml^−1^). After stirring for 6 h, the black powder was collected by centrifugation and the precipitated powder was washed with isopropanol to remove the excess TIP. The subsequent hydrolysis of TIP was initiated by exposure to air, leading to the conversion of TIP into TiO_2_. This impregnation/washing cycle was repeated twice in order to completely fill carbon frameworks with TiO_2_. After drying, the product was heated at 350 °C in argon for 1 h to crystallize the embedded TiO_2_ NPs.

### Preparation of Ti_x_Sn_1−x_O_2_ NP superlattices

A homogeneous mixture of TIP and SnCl_2_ with different molar ratios dissolved in ethanol was used as the precursor for the growth of Ti_*x*_Sn_1−*x*_O_2_ NP superlattices. In a typical synthesis of Ti_0.3_Sn_0.7_O_2_ NP superlattices, 0.4 mmol of TIP and 0.6 mmol of SnCl_2_ were dissolved in 1 ml of ethanol to form a homogeneous solution, into which 5 mg of dried mesoporous carbon powder was added with stirring. After 24 h, the product was centrifuged and washed with ethanol to remove the excess precursor. The precursor hydrolysis was initiated by the addition of ammonium hydroxide. This impregnation/washing cycle was repeated twice in order to completely load carbon frameworks with Ti_0.3_Sn_0.7_O_2_ NPs. After drying, the product was heated at 350 °C in argon for 1 h to crystallize the embedded Ti_0.3_Sn_0.7_O_2_ NPs.

### Preparation of zirconium phosphate NP superlattices

Zirconium phosphate (ZrP) NP superlattices were prepared by using a homogeneous mixture of ZrCl_4_ and triethyl phosphate as the precursor. Briefly, 0.45 mmol of ZrCl_4_ and 0.45 mmol of triethyl phosphate were mixed in 2 ml of ethanol to form a homogeneous solution, into which 5 mg of dried mesoporous carbon powder was added with vigorous stirring for 2 h. After that, the product was centrifuged and washed with ethanol to remove the excess precursor. The precipitated powder was then put in an oven at 110 °C for 2 h to induce the formation of ZrP NP superlattices.

### Synthesis of 13-nm SnO_2_ NPs in control experiments

In control experiments, colloidal SnO_2_ NPs with a similar size as our SnO_2_ NP superlattices were synthesized by a literature method^45^. In a typical synthesis, 10 ml of cationic surfactant (CTAB) solution (0.08 mol l^−1^) was mixed with 10 ml of NH_3_.H_2_O (25% aqueous solution) to form a homogeneous solution, into which 4.65 g of SnCl_4_ was added under vigorous stirring. After 4 h, the product was filtered and washed with distilled water for several times. The product was then heated at 400 °C in air for 2 h to increase the crystallinity, leading to SnO_2_ NPs with a mean diameter of 13 nm. Sucrose was used as the carbon source for the formation of carbon-coated SnO_2_ nanocomposite. In a typical preparation of SnO_2_/C nanocomposite with a carbon content of~26 wt%, 0.35 g of SnO_2_ NPs was mixed with a sucrose solution (0.4 g in 5 ml H_2_O), and the resulting mixture was pre-heated at 180 °C in an oven for 3 h. The dried powder was then heated in a quartz furnace at 500 °C for 5 h in argon, resulting into carbon-coated SnO_2_ nanocomposite.

### Instrumentation

TEM images, HRTEM images, scanning TEM images, elemental mapping, and EDS spectra were obtained using a Tecnai G2 20 TWIN microscope operated at 200 kV. SEM images and EDS spectra were recorded using a Zeiss Ultra-55 microscope operated at 5 and 10 kV, respectively. XRD was carried out on a Bruker D4 X-ray diffractometer, while SAXS was performed on a Nanostar U small-angle X-ray scattering system using Cu Kα radiation (40 kV, 35 mA). Nitrogen adsorption–desorption isotherms were recorded on a Tristar 3000 instrument. Before measurements, the samples were degassed at 300 °C for 5 h. Raman spectra were collected at room temperature on an XploRA Raman system. TGA measurements were carried out on a Perkin–Elmer Pyris 1 thermogravimetric analyzer.

### Electrochemical measurements

The battery performance was evaluated by galvanostatic cycling of 2025-type coin cells assembled in an argon-filled glove box, with SnO_2_ NP superlattices as the working electrode and lithium foil as the counter and reference electrode. The electrolyte was a 1.0 M LiPF_6_ solution in a mixture of ethylene carbonate, dimethyl carbonate and ethyl methyl carbonate (1:1:1 in volume), and a polypropylene film (Celgard-2300) was used as the separator. The working electrodes were prepared by a slurry-coating procedure. The slurry consisted of SnO_2_ NP superlattices, acetylene black (Super P) and polyvinylidene fluoride binder with a mass ratio of 7:2:1 dissolved in *N*-methyl-2-pyrrolidinone. This slurry was spread on a copper foil, which acted as a current collector. The electrodes were dried at 90 °C for 4 h in air, and then at 90 °C in vacuum for another 12 h. Cyclic voltammetry was carried out on an electrochemical workstation (Autolab 204 N), while galvanostatic measurements were performed on a Neware cell test instrument, which was cycled between 0.005 and 3.00 V (versus Li/Li^+^) at various current densities.

## Author contributions

Y.J. and A.D. conceived and designed the experiments. Y.J. and D.H. performed the experiments. Y.D., X.Z., G.G. and J.H. collected and analysed the XRD and TEM data. Y.J., D.Y. and A.D. wrote the paper. All authors discussed the results and commented on the manuscript.

## Additional information

**How to cite this article:** Jiao, Y. *et al*. Fabrication of three-dimensionally interconnected nanoparticle superlattices and their lithium-ion storage properties. *Nat. Commun.* 6:6420 doi: 10.1038/ncomms7420 (2015).

## Supplementary Material

Supplementary Information Supplementary Figures 1-13 and Supplementary Note 1
