# Supplementary material for: Fabrication of three-dimensionally interconnected nanoparticle superlattices and their lithium-ion storage properties
Source: Nat Commun. 2015 Mar 3;6:6420. doi: 10.1038/ncomms7420 (PMC4366534; doi:10.1038/ncomms7420)
Supplement: Supplementary Information — Supplementary Figures 1-13 and Supplementary Note 1 [file ncomms7420-s1.pdf]

Supplementary Figures

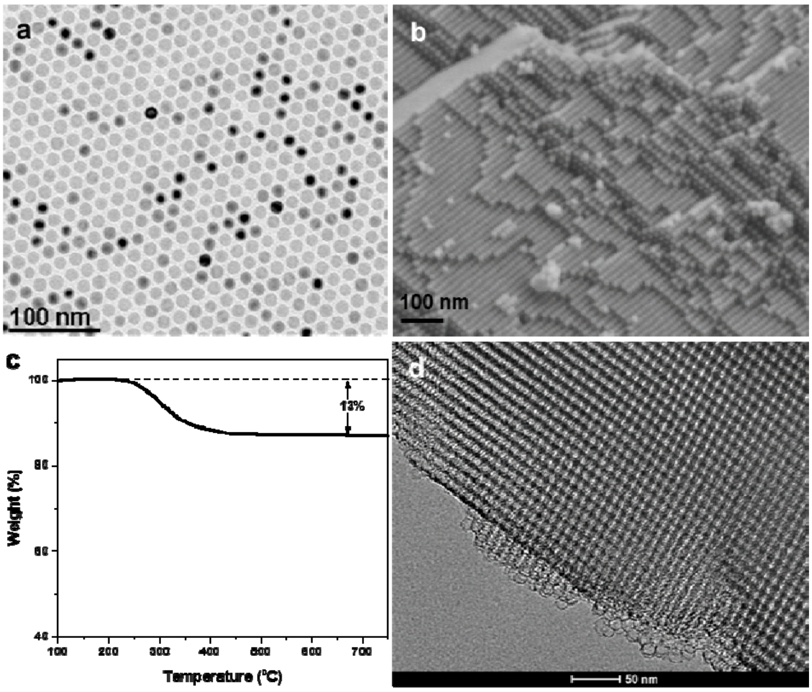

Supplementary Figure 1. **a**, TEM image of 11-nm Fe_3O_4 NPs used in this study. **b**, HRSEM image of Fe_3O_4 NP superlattices formed in the absence of squalane. **c**, TGA scan of Fe_3O_4 NP superlattices shown in (**b**), which was carried out in air at a heating rate of 20 ^{o}C/min. The oleic acid content is determined to be ~ 15.9 wt% (see Supplementary Note 1). **d**, TEM image of ordered mesoporous carbon frameworks derived from Fe_3O_4 NP superlattices shown in (**b**), suggesting that squalane is not critical for the formation of mesoporous carbons.

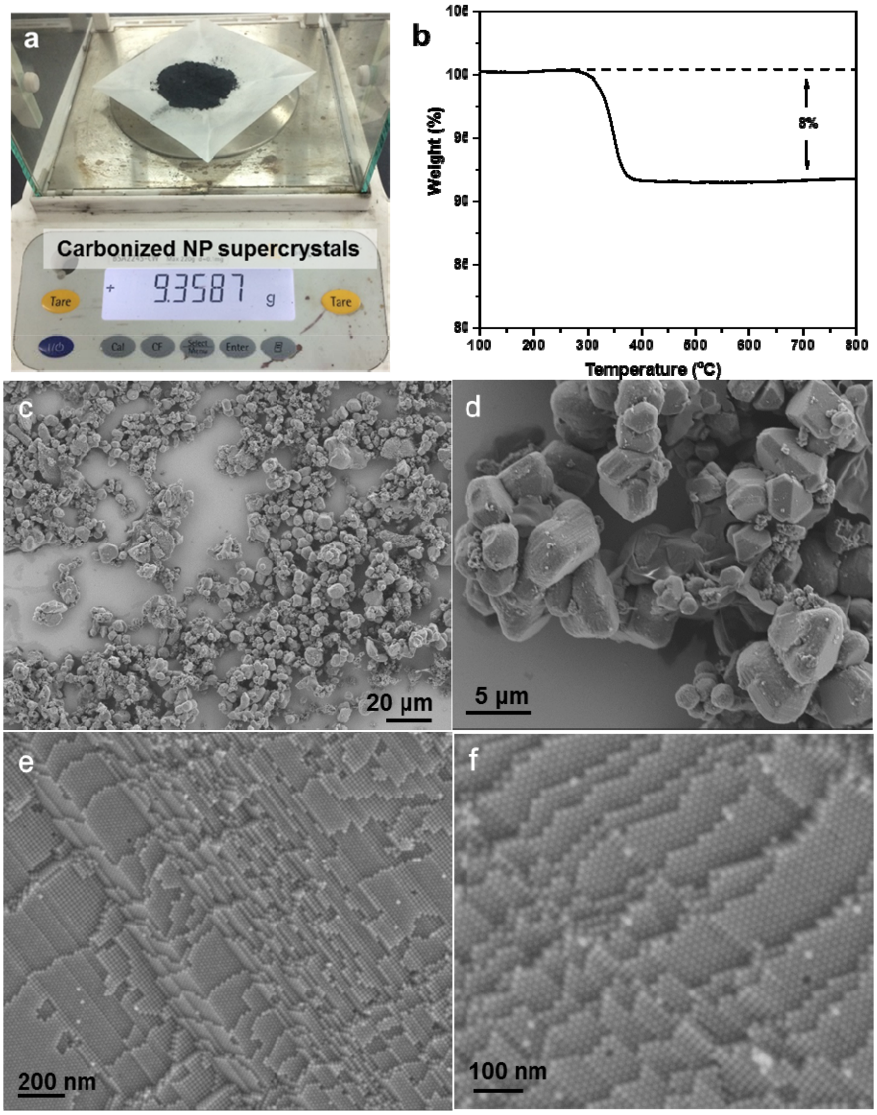

Supplementary Figure 2. a, Photograph of carbonized Fe_3O_4 NP supercrystals. **b**, TGA scan of carbonized Fe_3O_4 NP supercrystals performed in air, showing the weight fraction of carbon species is 1 − (92%/159.6)×(2/3)×231.5 = ~ 11 wt%, considering the oxidation of Fe_3O_4 into Fe_2O_3. **c**,**d**, Low-magnification SEM images of carbonized Fe_3O_4 NP supercrystals. **e**,**f**, HRSEM images of carbonized Fe_3O_4 NP supercrystals. The well retained crystal-like macroscopic morphology and the long-range NP ordering suggest that cracks and other structural defects are largely prevented during heat treatment of Fe_3O_4 NP supercrystals.

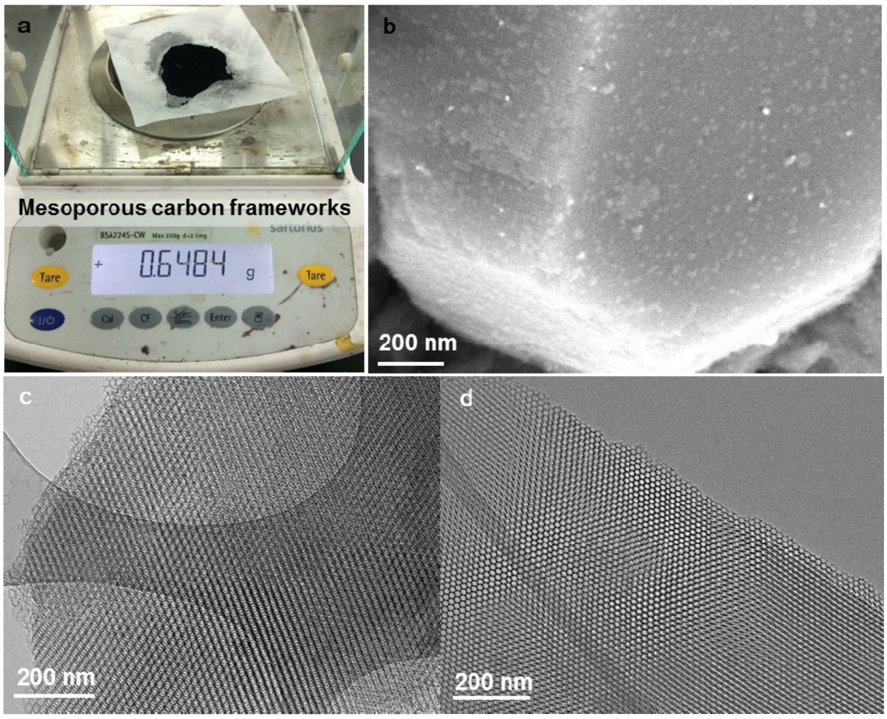

Supplementary Figure 3. a, Photograph of mesoporous carbon frameworks. HRSEM (**b**) and low-magnification TEM (**c**,**d**) images of mesoporous carbon frameworks, showing the highly ordered porous structure.

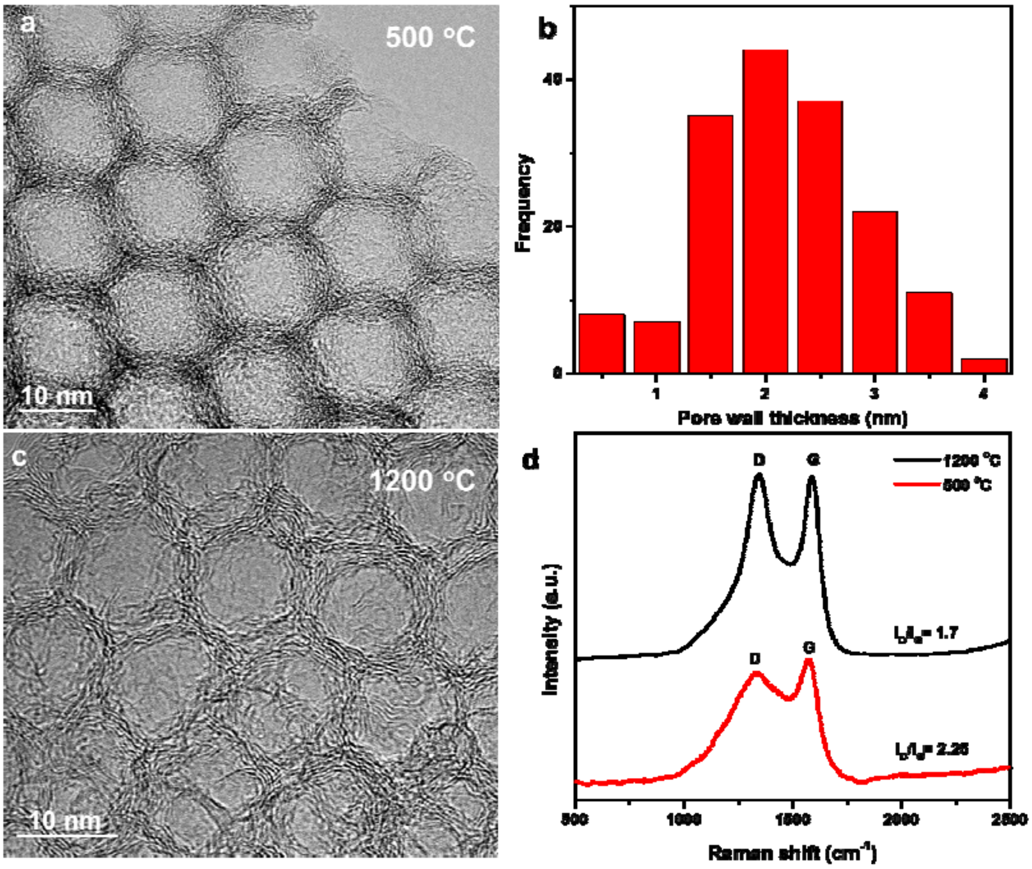

Supplementary Figure 4. **a**, HRTEM image of mesoporous carbon frameworks, showing the partially graphitic feature of the pore walls. **b**, Pore wall thickness distribution histogram, showing the mean wall thickness is 2 nm. **c**, HRTEM image of carbon frameworks after thermal treatment at 1200 ^{o}C in argon, showing the increased graphitization degree of the pore walls. **d**, Raman spectra of carbon frameworks treated at different temperatures. The D and G band intensity ratios are included to indicate the respective graphitization degree.

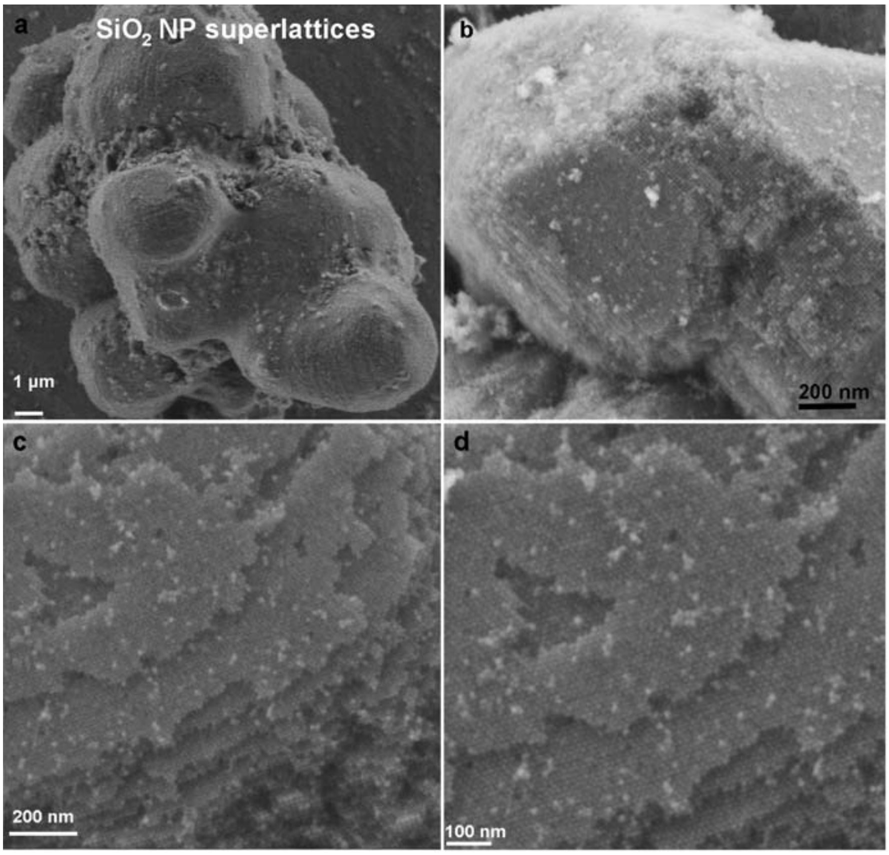

Supplementary Figure 5. Low- (**a**) and high-magnification (**b**) SEM images of SiO_2 NP superlattices, showing the general appearance of the sample. **c**,**d**, HRSEM images of SiO_2 NP superlattices, showing the high degree of NP ordering.

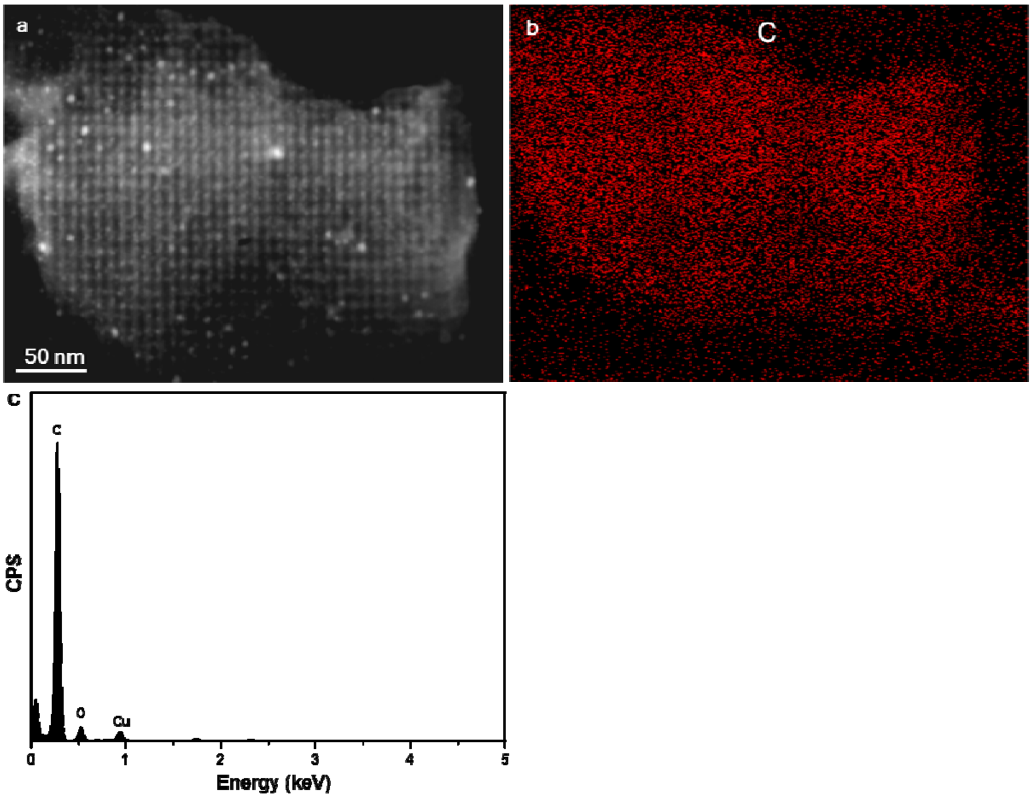

Supplementary Figure 6. Scanning TEM image (**a**), elemental mapping (**b**), and the corresponding EDS spectrum (**c**) of 3D carbon NP superlattices. The Cu signal in (**c**) is from the TEM grid.

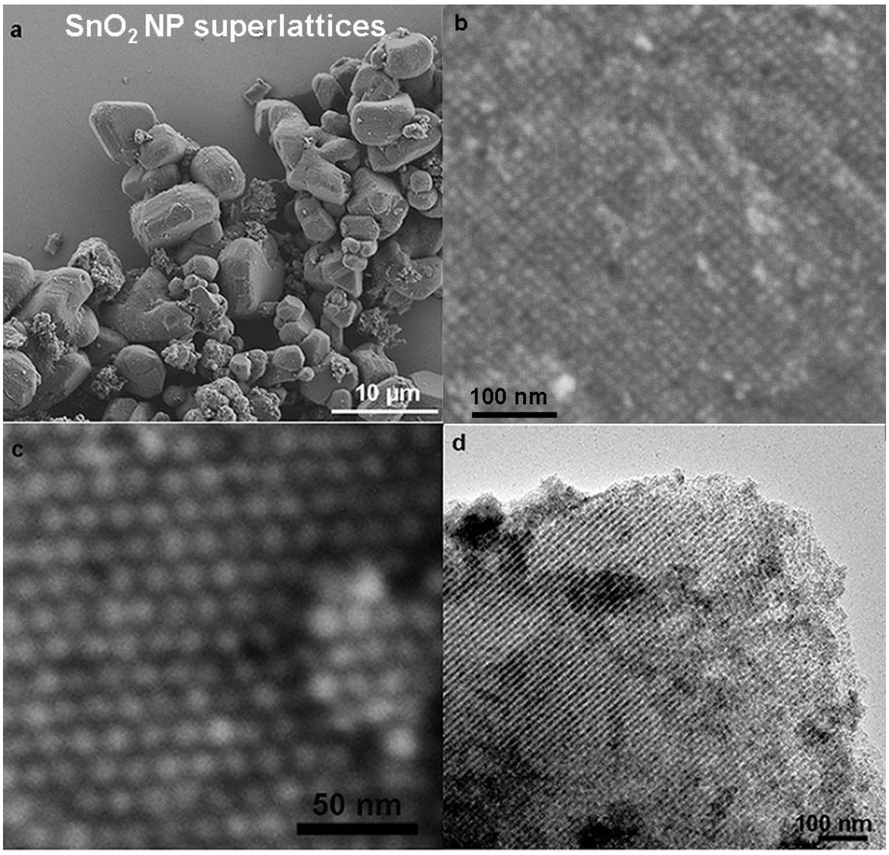

Supplementary Figure 7. Low- (**a**) and high-magnification (**b**) SEM images of SnO_2 NP superlattices, showing the general appearance of the sample. HRSEM (**c**) and TEM (**d**) images of SnO_2 NP superlattices, showing the high degree of NP ordering.

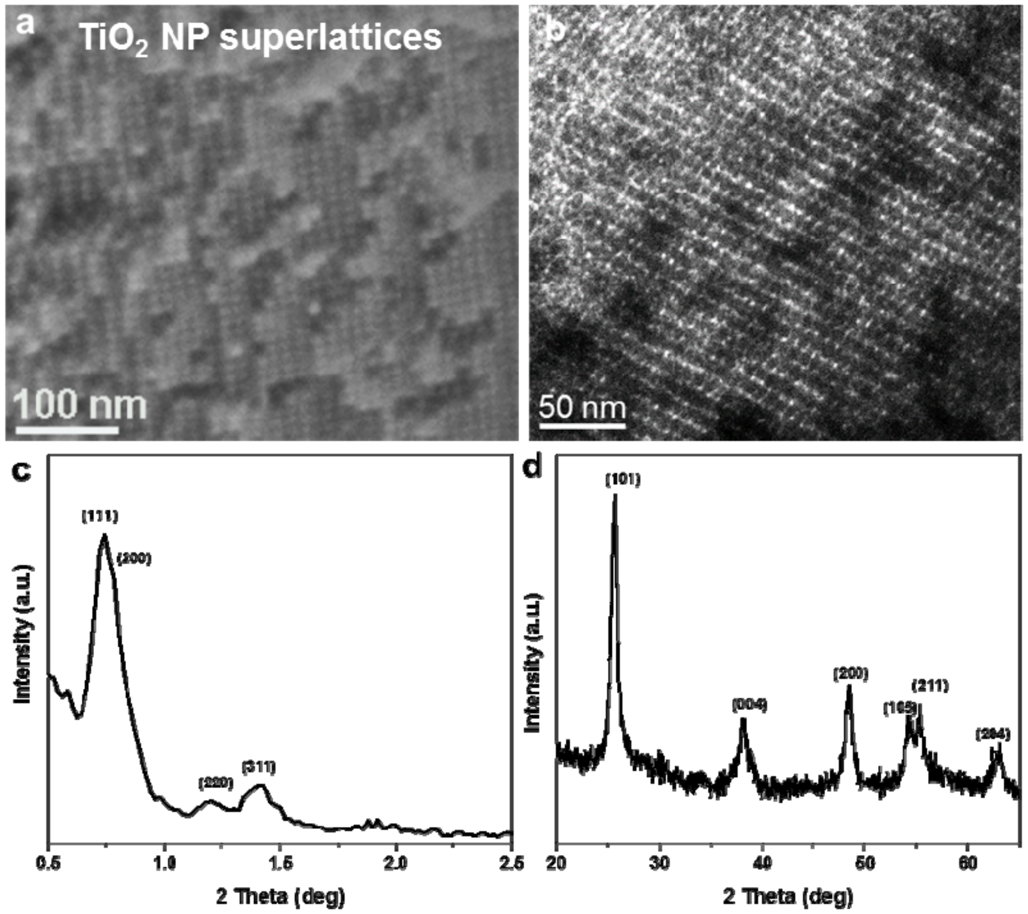

Supplementary Figure 8. SEM (**a**) and TEM (**b**) images of TiO_2 NP superlattices. **c**, SAXS pattern of TiO_2 NP superlattices, showing the *fcc* superlattice structure. **d**, XRD pattern of TiO_2 NP superlattices. The well resolved peaks suggest that the embedded TiO_2 NPs possess a high crystallinity with an anatase crystal structure.

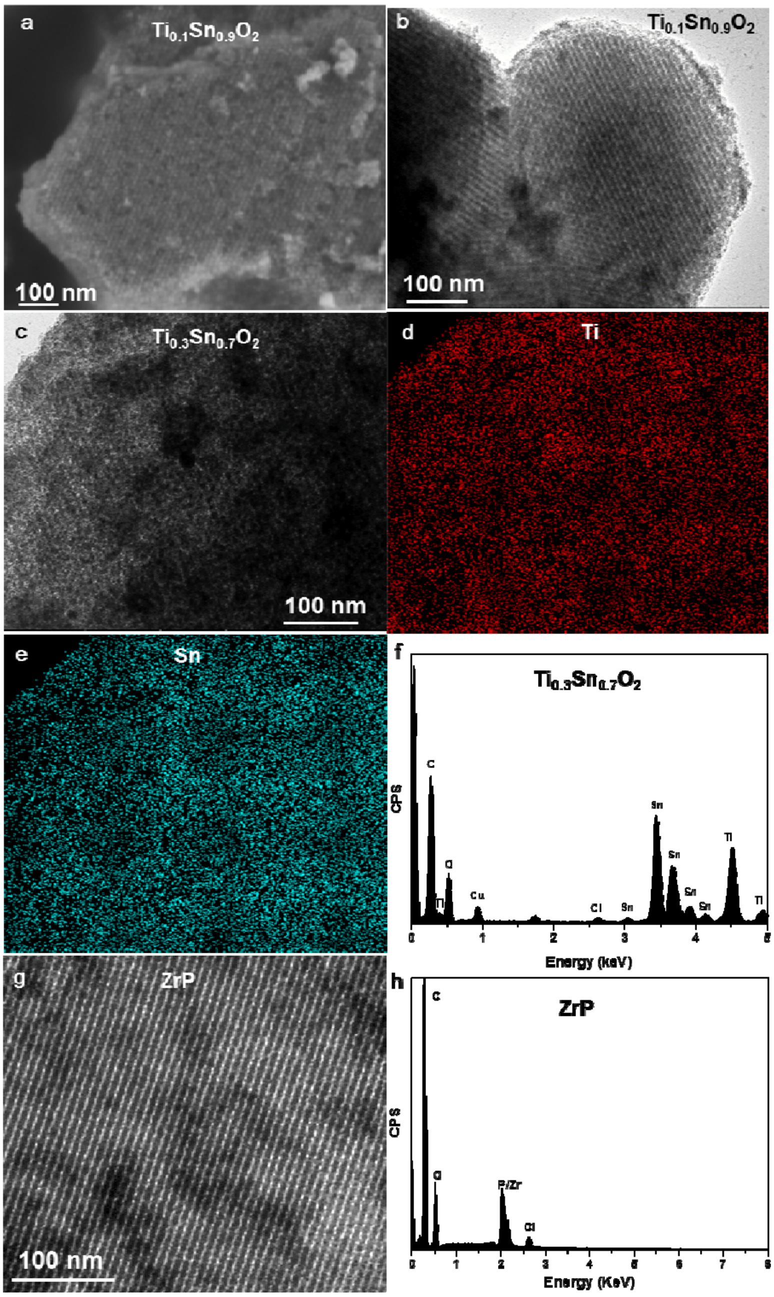

Supplementary Figure 9. Low-magnification SEM (**a**) and TEM (**b**) images of $Ti_{0.1}Sn_{0.9}O_2$ NP superlattices, showing the general appearance of the sample. TEM image (**c**), EDS elemental mapping (**d**,**e**), and the corresponding EDS spectrum (**f**) of $Ti_{0.3}Sn_{0.7}O_2$ NP superlattices. The uniform distribution of Ti and Sn indicates that the homogenous filling of $Ti_{0.3}Sn_{0.7}O_2$ NPs within the carbon framework. TEM image (**g**) and EDS spectrum (**h**) of zirconium phosphate (ZrP) NP superlattices. The presence of Cl in (**f**,**h**) is resulted from HCl etching.

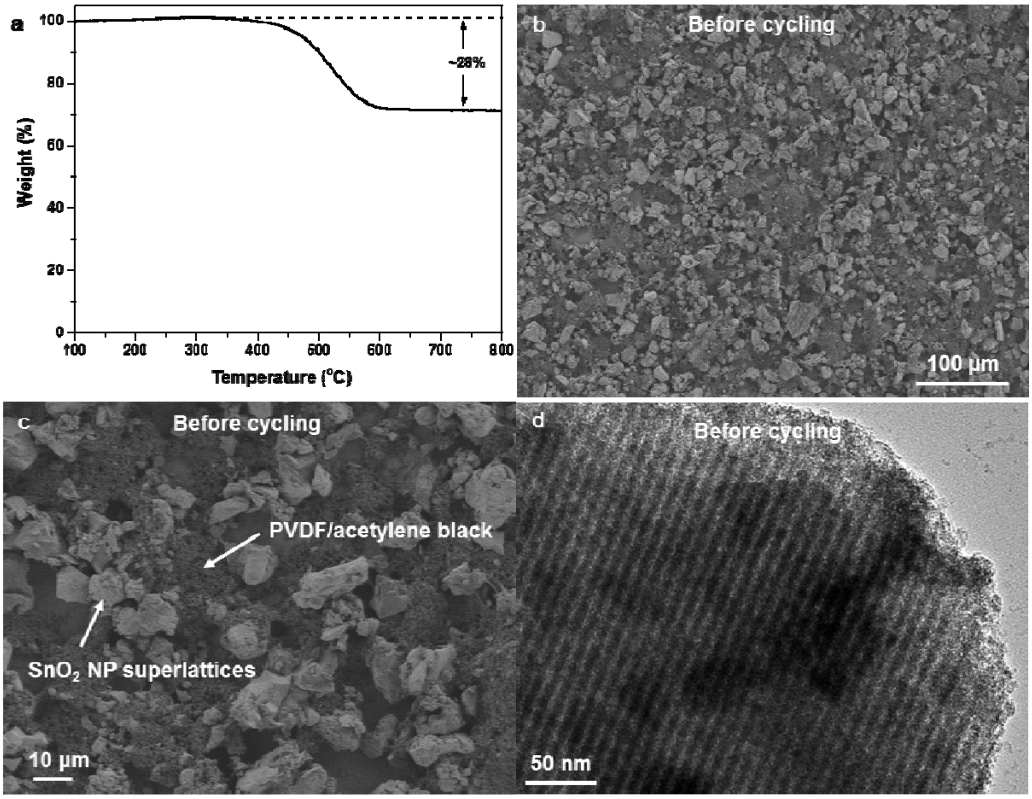

Supplementary Figure 10. a, TGA scan of SnO_2 NP superlattices used for LIB anodes, which was carried out in air at a heating rate of 20 °C/min. The carbon content is determined to be ~ 28 wt%. Low- (**b**) and high-magnification (**c**) SEM images of a typical SnO_2 NP superlattice anode before cycling, showing the homogeneous distribution of SnO_2 NP superlattices in the electrode. **d**, TEM image of a SnO_2 NP superlattice anode before cycling, showing the intact superlattice structure during electrode preparation.

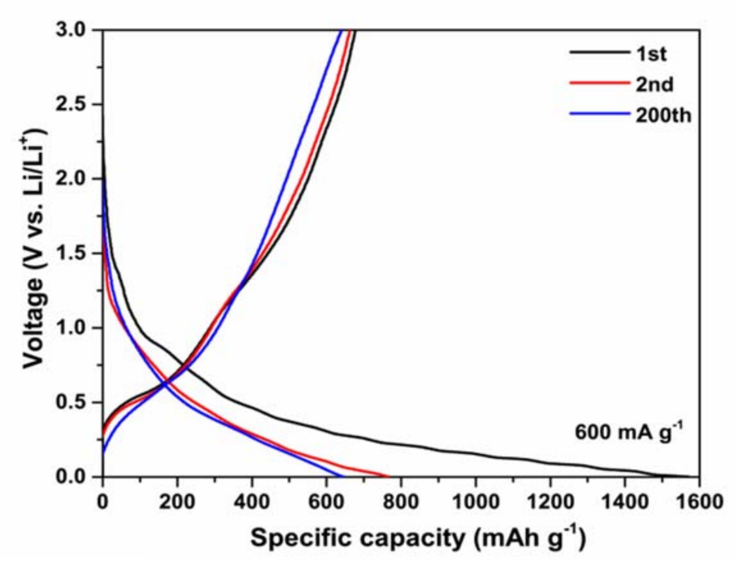

Supplementary Figure 11. Galvanostatic charge/discharge voltage profiles of SnO_2 NP superlattices for the 1st, 2nd, and 200th cycles at a current density of 600 mA g^{-1}.

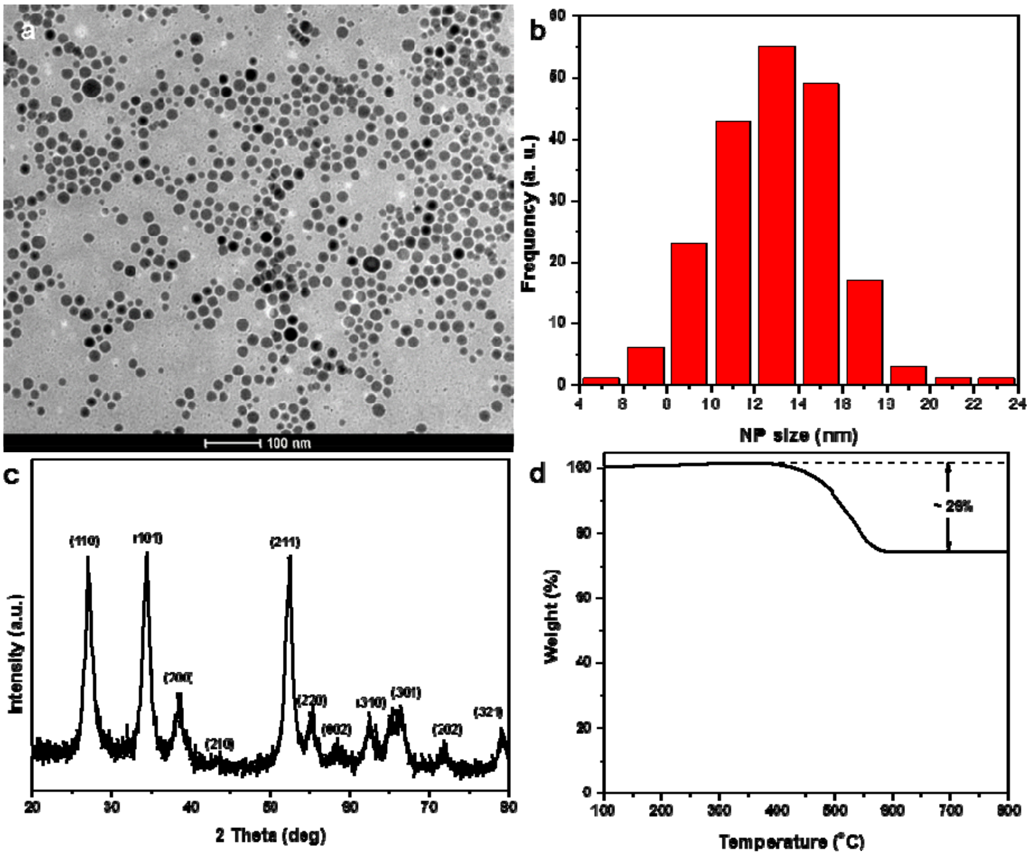

Supplementary Figure 12. Representative TEM image (**a**), size distribution histogram (**b**), and XRD pattern (**c**) of colloidal SnO_2 NPs with an average diameter of 13 nm, which were used to fabricate LIB anodes in control experiments. **d**, TGA scan of SnO_2/C nanocomposite, which was carried out in air at a heating rate of 20 ^oC/min. The carbon content is determined to be ~ 26 wt%, close to that (~ 28 wt%) of SnO_2 NP superlattices.

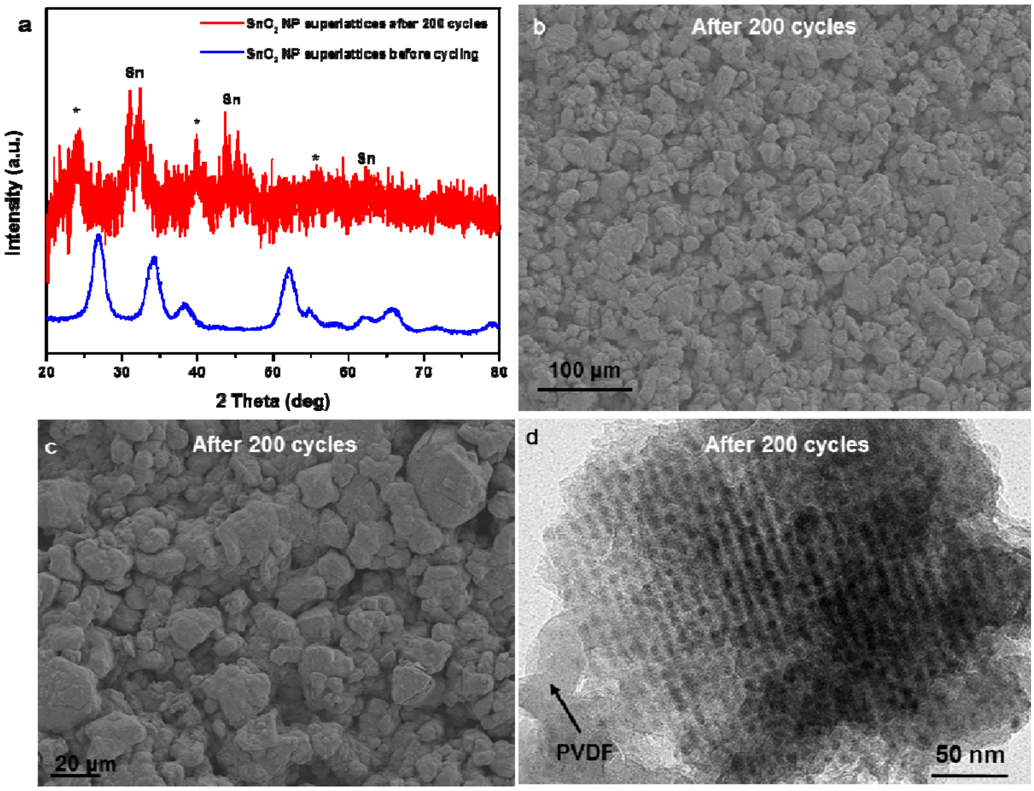

Supplementary Figure 13. a, XRD pattern of SnO_2 NP superlattices after 200 cycles at a current density of 600 mA g^{-1}, showing the conversion of SnO_2 into Sn. The peaks indicated by the asterisks are ascribed to PVDF. For comparison, the XRD pattern of SnO_2 NP superlattices before cycling is also included. Low- (**b**) and high-magnification (**c**) SEM images of a typical SnO_2 NP superlattice anode after 200 cycles, showing the retained secondary particle morphology. **d**, TEM image of SnO_2 NP superlattices after 200 cycles, showing the preservation of ordered structure without NP aggregation.

Supplementary Notes

Supplementary Note 1. The surface coverage (ϕ) of oleic acid (OA) ligands can be calculated by $\phi = \frac{N_{OA}}{SA_{NP}}$, where N_{OA} is the number of OA molecules per Fe_3O_4 NP and SA_{NP} is the surface area of a single Fe_3O_4 NP. The mass of a single Fe_3O_4 NP (density = 5.18 g/cm^3) with a diameter of 11 nm is $(4\pi/3)\times(5.5\times10^{-7})^3\times5.18 = 3.61\times10^{-18}$ g. Since Fe_3O_4 NPs are oxidized into Fe_2O_3 when heated in air, the Fe_3O_4 content determined from TGA in **Supplementary Fig. 1c** is $(87\%/159.6)\times(2/3)\times231.5 = 84.1$ wt%. Therefore, the actual OA content in Fe_3O_4 NP superlattices shown in **Supplementary Fig. 1b** should be 1-84.1% = 15.9 wt%. The number of OA molecules (molecular weight = 282.4 g/mol) per gram of Fe_3O_4 NP superlattices is $(0.159/282.4)\times6.02\times10^{23} = 3.39\times10^{20}$, while the number of Fe_3O_4 NPs per gram of Fe_3O_4 NP superlattices is $0.841/(3.61\times10^{-18}) = 2.33\times10^{17}$. As a result, the number of OA molecules per Fe_3O_4 NP is $N_{OA} = 3.39\times10^{22}/2.33\times10^{19} = \sim 1455$. Given that the surface area of a single Fe_3O_4 NP is $4\pi\times5.5^2 = 379.9$ nm^2, the area ligand coverage is $\phi = 1189/379.9 = 3.83$ nm^{-2}, i.e., 3.83 OA molecules per nm^2. This OA density is enough to form mesoporous carbon frameworks as shown in **Supplementary Fig. 1d** upon carbonization and acid etching.
